# Supplementary material for: Cryo-EM Structure of the 2019-nCoV Spike in the Prefusion Conformation
Source: bioRxiv. 2020 Feb 15:2020.02.11.944462. Preprint. [Version 1] doi: 10.1101/2020.02.11.944462 (PMC7217118; doi:10.1101/2020.02.11.944462)
Supplement: 1 [file NIHPP2020.02.11.944462-supplement-1.pdf]

bioRxiv preprint doi: https://doi.org/10.1101/2020.02.11.944462. The copyright holder for this preprint (which was not peer-reviewed) is the author/funder. It is made available under a CC-BY 4.0 International license.

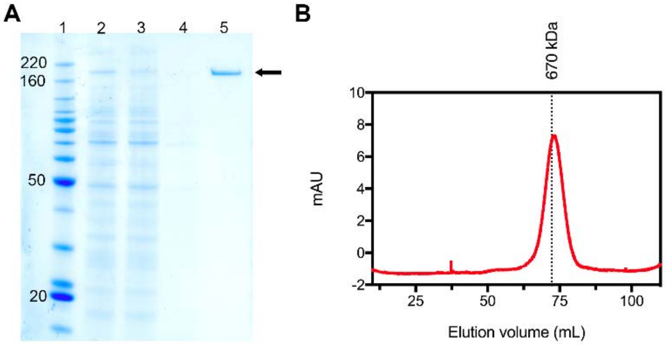

Supplementary Figure 1. 2019-nCoV S expression and purification. (**A**) SDS-PAGE analysis of the 2019-nCoV S protein. Lane 1: molecular weight ladder, with relevant bands labeled in kilodaltons (*left*); lane 2: filtered supernatant from transfected cells; lane 3: supernatant after passing through StrepTactin resin; lane 4: wash of StrepTactin resin; lane 5: elution from StrepTactin resin. The band corresponding to 2019-nCoV S is denoted with a black arrow. (**B**) Size-exclusion chromatogram of the affinity-purified 2019-nCoV S protein. Data from a Superose 6 10/300 column are shown in red. The elution volume of a 670 kilodalton molecular weight standard is shown as a black dotted line.

bioRxiv preprint doi: https://doi.org/10.1101/2020.02.11.944462. The copyright holder for this preprint (which was not peer-reviewed) is the author/funder. It is made available under a CC-BY 4.0 International license.

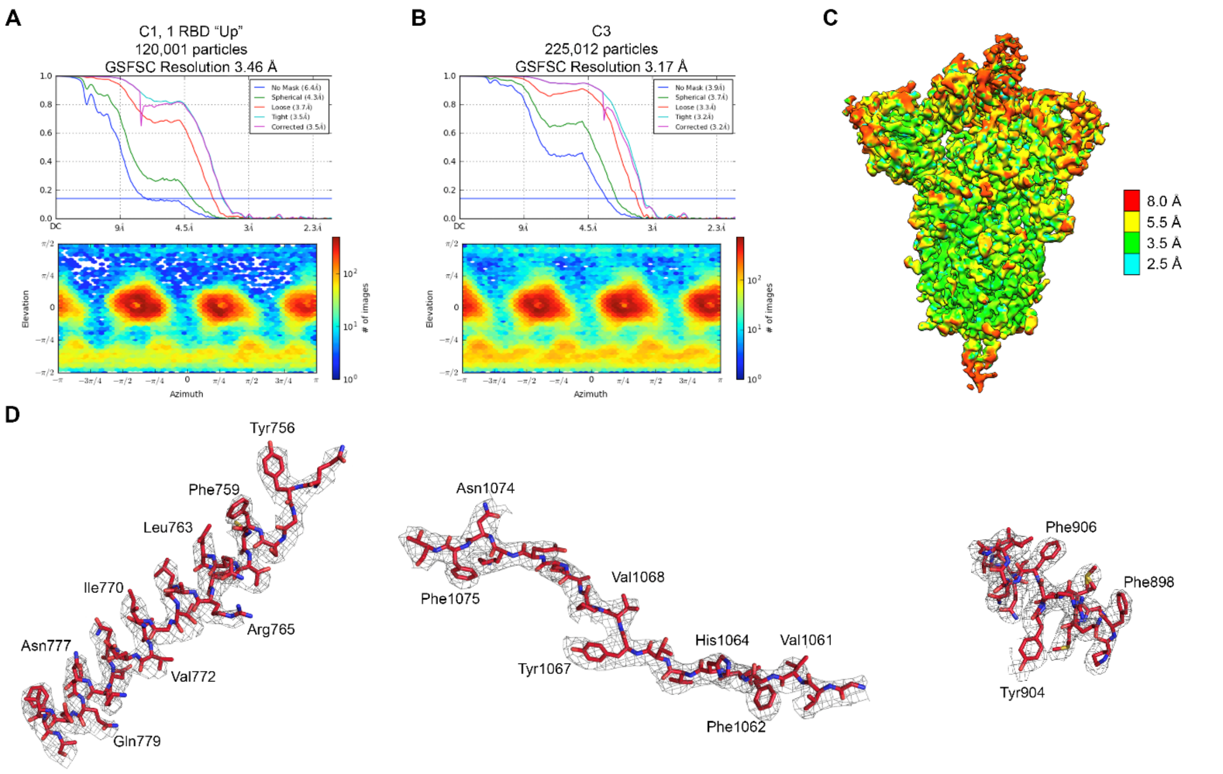

Supplementary Figure 2. **Cryo-EM structure validation**. (**A**) FSC curves (*top*) and the viewing direction distribution plot (*bottom*) for 2019-nCoV S with a single RBD "up". (**B**) FSC curves (*top*) and the viewing direction distribution plot (*bottom*) for the 2019-nCoV S processed with C3 symmetry. (**C**) The cryo-EM density of the 2019-nCoV S with a single RBD "up" is shown, colored according to local resolution. (**D**) Density from S2 of the C3-refined 2019-nCoV S structure. Residues are shown as sticks, colored according to **Figure 1A** with oxygen atoms colored red, nitrogens colored blue and sulfurs colored yellow. The cryo-EM density map is shown as a gray mesh.

bioRxiv preprint doi: https://doi.org/10.1101/2020.02.11.944462. The copyright holder for this preprint (which was not peer-reviewed) is the author/funder. It is made available under a CC-BY 4.0 International license.

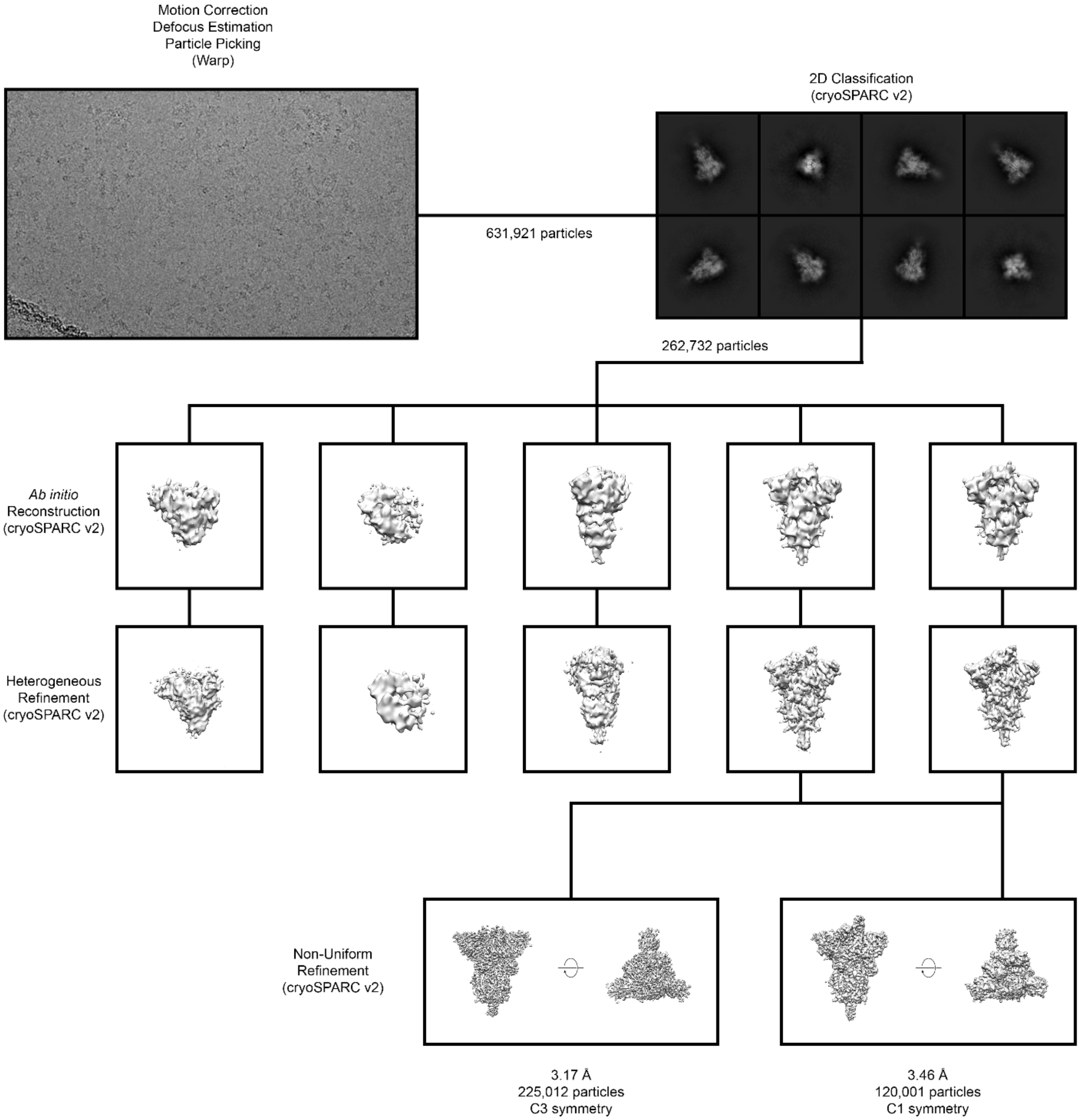

Supplementary Figure 3. Cryo-EM data processing workflow.

bioRxiv preprint doi: https://doi.org/10.1101/2020.02.11.944462. The copyright holder for this preprint (which was not peer-reviewed) is the author/funder. It is made available under a CC-BY 4.0 International license.

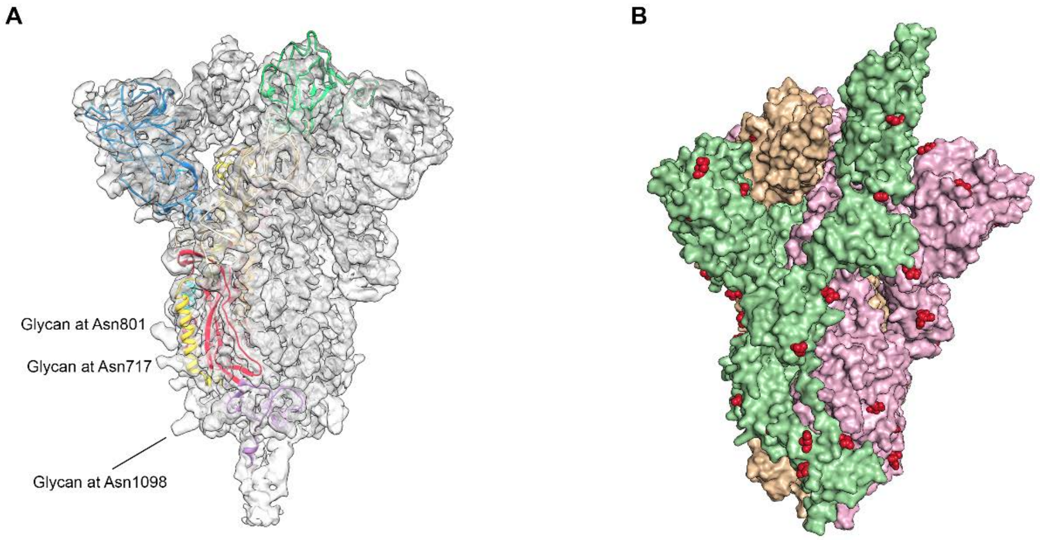

Supplementary Figure 4. **Cryo-EM map and *N*-linked glycosylation sites**. (**A**) The unsharpened cryo-EM density map for the C3-processed 2019-nCoV S is shown as a transparent molecular surface, with a single protomer fit into the map shown in ribbons and colored according to **Figure 1A**. Some S2 density that corresponds to *N*-linked glycans is labeled. (**B**) The 2019-nCoV S trimer is shown as a molecular surface with each protomer colored green, pink or tan. Asparagine residues that correspond to *N*-linked glycosylation sites are shown as red spheres.

bioRxiv preprint doi: https://doi.org/10.1101/2020.02.11.944462. The copyright holder for this preprint (which was not peer-reviewed) is the author/funder. It is made available under a CC-BY 4.0 International license.

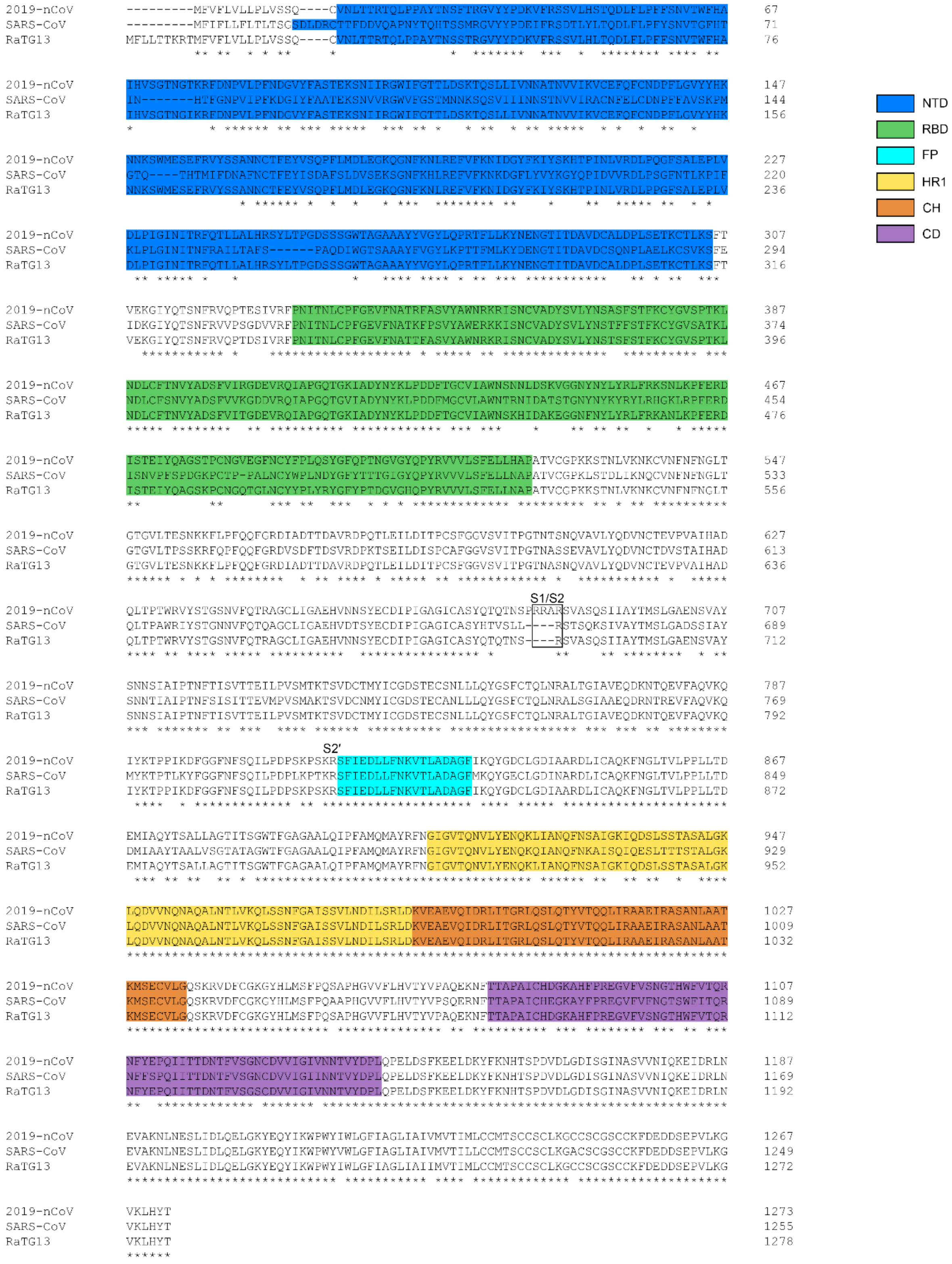

Supplementary Figure 5. **Sequence alignment of 2019-nCoV S, SARS-CoV S and RaTG13 S**. Identical residues are denoted by an "*" beneath the consensus position. Structural domains are colored according to **Figure 1A**.

bioRxiv preprint doi: https://doi.org/10.1101/2020.02.11.944462. The copyright holder for this preprint (which was not peer-reviewed) is the author/funder. It is made available under a CC-BY 4.0 International license.

A

Domain	Variant residues
NTD	Phe32, Ser50, Thr76, Gln218
RBD	Arg346, Ala372, Arg403, Asn439, Asn440, Leu441, Ser443, Val445, Tyr449, Ser459, Thr478, Val483, Glu484, Phe486, Phe490, Gln493, Ser494, Gln498, Asn501, Tyr505, His519
SD1	Glu324
SD2	Thr604
S1/S2	682-PRRA-685 (insertion)
S2	Asn1125, Val1228

B

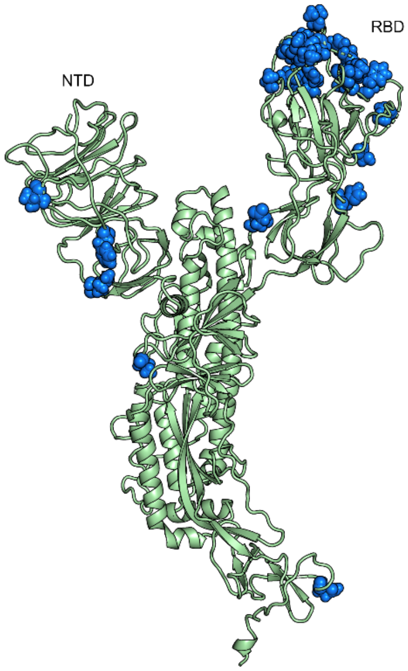

C

Domain	Substitution	2019-nCoV isolates
NTD	F32I	IVDC-HB-01/2019
	H49Y	20SF028/2020, 20SF040/2020, 20SF174/2020
	S247R	VIC01/2020
RBD	N354D	SZTH-004/2020
	D364Y	SZTH-004/2020
	V367F	IDF0372/2020, IDF0373/2020
SD2	D614G	BavPat1/2020
S2	V1129L	SZTH-001/2020
	E1262G	SZTH-001/2020

D

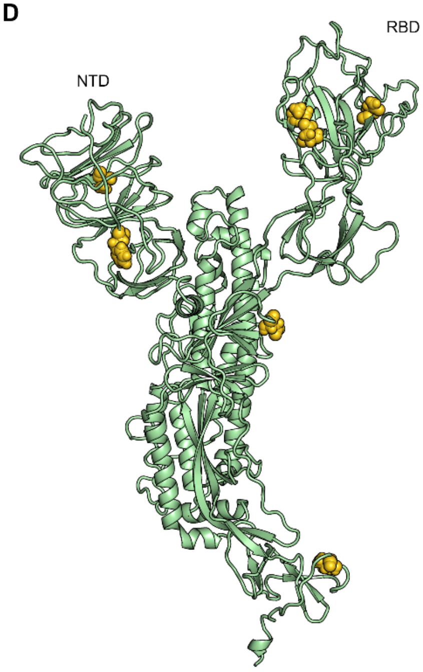

Supplementary Figure 6. **Sequence variability between RaTG13 S and 2019-nCoV S clinical isolates**. (**A**) Table shows residues in the 2019-nCoV S protein that vary in RaTG13, grouped by structural domain. (**B**) A single monomer of the 2019-nCoV S protein is shown in ribbons, colored green. RaTG13 variant residues are shown as blue spheres. (**C**) Table shows variations in the 2019-nCoV S sequence based on 61 clinical isolates and the domains wherein these variations occur. (**D**) A single monomer of the 2019-nCoV S protein is shown in ribbons, colored green. Variant residues are shown as gold spheres.

bioRxiv preprint doi: https://doi.org/10.1101/2020.02.11.944462. The copyright holder for this preprint (which was not peer-reviewed) is the author/funder. It is made available under a CC-BY 4.0 International license.

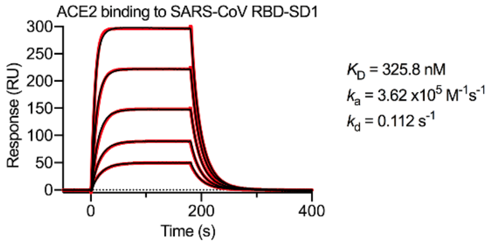

Supplementary Figure 7. **SARS-CoV RBD-SD1 binding to human ACE2**. An SPR sensorgram is shown, displaying the binding between soluble human ACE2 and immobilized SARS-CoV RBD-SD1. The data are shown as black lines and the best fit of the data to a 1:1 binding model is shown in red.

bioRxiv preprint doi: https://doi.org/10.1101/2020.02.11.944462. The copyright holder for this preprint (which was not peer-reviewed) is the author/funder. It is made available under a CC-BY 4.0 International license.

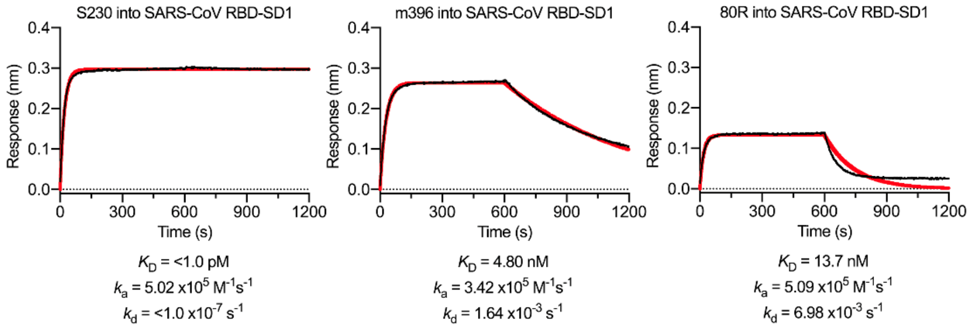

Supplementary Figure 8. **SARS-CoV RBD-directed antibody validation**. The monoclonal antibodies that were tested for cross-reactivity to the 2019-nCoV RBD-SD1 were also tested for binding to the SARS-CoV S RBD-SD1 as a positive control. Binding data are shown as a black line and the best fit of the data to a 1:1 binding model is shown in red.

bioRxiv preprint doi: https://doi.org/10.1101/2020.02.11.944462. The copyright holder for this preprint (which was not peer-reviewed) is the author/funder. It is made available under a CC-BY 4.0 International license.

Supplementary Table 1. Cryo-EM data collection and refinement statistics.

EM data collection and reconstruction statistics

Protein	2019-nCoV S 1 RBD "up"	2019-nCoV S C3 symmetry
EMDB	EMD-21375	EMD-21374
Microscope	FEI Titan Krios	FEI Titan Krios
Voltage (kV)	300	300
Detector	Gatan K3	Gatan K3
Exposure (e^-/Å^2)	36	36
Defocus range (μm)	0.8–2.8	0.8–2.8
Final particles	120,001	225,012
Symmetry imposed	n/a (C1)	C3
Resolution (Å)	3.46	3.17

Model refinement and validation statistics

PDB	6VSB
Composition	
Amino acids	2,905
Glycans	61
RMSD bonds (Å)	0.004
RMSD angles (º)	0.88
Ramachandran	
Favored (%)	94.6
Allowed (%)	5.2
Outliers (%)	0.2
Rotamer outliers (%)	0.64
Clash score	12.8
MolProbity score	1.99

bioRxiv preprint doi: https://doi.org/10.1101/2020.02.11.944462. The copyright holder for this preprint (which was not peer-reviewed) is the author/funder. It is made available under a CC-BY 4.0 International license.

Supplementary Movie 1. CryoSPARC 3D variability analysis. 2019-nCoV S trimer viewed from the side, along the viral membrane.

Supplementary Movie 2. CryoSPARC 3D variability analysis. 2019-nCoV S trimer viewed from the top, toward the viral membrane.
